# Proportion of Preterm birth and associated factors among mothers who gave birth in Debretabor town health institutions, northwest, Ethiopia

**DOI:** 10.1186/s13104-018-4037-7

**Published:** 2019-01-03

**Authors:** Dawit Gebeyehu Mekonen, Ayenew Engida Yismaw, Tewodros Siyoum Nigussie, Worku Mequanint Ambaw

**Affiliations:** College of Medicine and Health Science, School of Midwifery, University of Gondar, Gondar, Ethiopia

**Keywords:** Preterm birth, Proportion, Debretabor Town

## Abstract

**Objective:**

Each year, about 15 million babies in the world are born too prematurely. Complication of preterm birth is the single largest direct cause of neonatal deaths and the second most common cause of under-5 deaths after infection. Therefore, assessing the proportion of preterm birth and associated factors among Mothers who gave Birth in Debretabor town health institutions have a paramount importance in designing an effective strategy to intervene.

**Result:**

In this study preterm birth was 12.8%. Obstetric complication [AOR = 6.6, 95% CI (3.4–12.6)], maternal Mid Upper Arm Circumference less than 24 cm [AOR = 2.6, 95% CI (1.1–6.1)], antenatal care follow up < 4 visits [AOR = 3.0, 95% CI (1.6–5.9)], being HIV positive [AOR = 5.1, 95% CI (1.7–15.4)], Premature Rupture Of membrane [AOR = 3.0, 95% CI (1.5–6.2)], and being Anemic [AOR = 2.9, 95% CI (1.3–6.6)] were found to be statistically significant. Proportion of preterm birth was high in Debretabor town. Timely identification of obstetric complications and health education to improve antenatal care utilization will minimize the proportion of preterm birth.

## Introduction

Preterm birth is defined by WHO as all viable births before 37 completed weeks of gestation or fewer than 259 days since the first day of woman’s last menstrual period [1]. Each year, more than one in 10 births, are born too prematurely in the world. Preterm birth is the single largest direct cause of neonatal deaths responsible for 35% and the second most common cause of under-5 deaths a year [2]. About 28% of early neonatal deaths are due to preterm birth [3].

Globally preterm neonates take the first place for neonatal intensive care unit (NICU) admission and longer hospital stay [4]. Complication of the preterm result in significant cost to the health sector, parents and the society. Therefore, the prediction and prevention of preterm birth is a major health care priority [5, 6].

Over 60% of preterm births occur in low- and middle-income countries (LMICs) and consistently risen in most countries [7]. Study on preterm birth highly recommends the need for focused and continuous studies across those nations in order to fill this information gap [8].

Ethiopia had reduced under-5 mortality by 67%, however, the reduction in neonatal mortality is not as impressive and premature birth is the leading cause accounts 37% [9].

Studies estimated preterm birth rates ranged from 5% in developed countries to 26% in developing countries [5]. In 2010 WHO estimated the global rates was 11.1%. From this majority (85%) occurred in sub-Saharan Africa and South Asia [5]. Another retrospective study estimated as 10.2% [10]. One cohort study estimated to be 15.8% [11], in Jeddah, Saudi Arabia, 13.7% [12], in Reliance Region (West of Algeria), 9.26% [13], in Brazilian young parturient women 21.7% and Multicenter Study in Brazil preterm births was 12.3%, ranging from 14.7% in the northeast region to 11.1% in the southeast [14, 15].

A cross-sectional study in the University of Ilorin teaching hospital, Ilorin, Nigeria, reported 11.8% [16], in Cameron and Malawi was 26.5% and 16.3% respectively [17, 18], and in the eastern part of Africa the largest prevalence of preterm was in Kenya 18.3% [19].

Studies conducted in different areas reported risk factors for preterm birth: low socio-economic status, malnutrition, pregnancy-related complications and history of preterm birth [20], age less than 20, PROM, UTI, multiple pregnancy, preeclampsia [15, 24], cervical insufficiency, fetal malformation, polyhydramnios, antepartum hemorrhage, previous abortion, high BMI, suffered from domestic violence [21], history of child death [22], residence, lack of antenatal care and maternal disease [23], null parity and being unmarried [25, 26]. Presence of chronic illness, the absence of antenatal follow up, and hematocrit (HCT) level < 33 were found to be significantly associated with preterm birth [27, 28].

Therefore determining factors has a great role in guiding health professionals and health policy makers to design the intervention strategy and applying necessary preventive and appropriate measures to decrease preterm birth.

## Main text

### Methods

#### Institutional based cross-sectional study design was conducted among mothers who gave birth in Debretabor town health institutions from June 1 to September 30, 2016

The source population was mothers who gave birth in Debretabor town health institutions and the study population was those mothers who gave birth during the study period.

By considering single population proportion formula the final sample sizes were 575. Health institutions providing labor and delivery service in the town were systematically selected. Then the sample for each health institution was proportionally allocated based on their patient flow prior to the data collection period. Systematic sampling technique was employed to select the study participants from each health institution. Neonates born at less than 37 completed weeks of gestation but after viability (28 weeks of gestation) were taken as preterm, gestational age was calculated based on LNMP or first-trimester ultrasound result. Maternal nutritional status was assessed by measuring the left middle upper arm circumference (MUAC) using non-stretchable world food program MUAC tapes. Most screening programs have used a cut off of 21–23 cm. Given that there is no international consensus on the cut off to use, a MUAC of < 24 cm was chosen for this study.

Data were collected by face to face interview and chart review using a structured and pre-tested questionnaire. Data were checked, coded and entered into EPI Info 7 and exported to SPSS 20 for analysis. Binary logistic regression was used to identify the associations between the dependent and independent variables. Those variables with a p-value of < 0.2 on binary logistic regression analysis were transferred to multivariable logistic regression. The degree of association was assessed using odds ratio with a 95% confidence interval and variables with a p-value < 0.05 were taken as statically significant.

### Results

#### Socio-demographic characteristics of respondents

A total of 548 participants were completed the interview making 95.3% of response rate. The mean age of the study participants was 27.7 with SD of 5.8. Sixteen years old was the minimum and 41 years old was the maximum ages of clients participated in this study. Majority of the participants were 517 (94.2%) Orthodox Christians in religion. Majority 538 (98.2%) of the respondents were married and one-third 184 (33.4%) of the mothers were secondary and above on their educational status (Table 1).

**Table 1:** Socio-demographic and economic characteristics of the women delivered in Debretabor town health institution northwest, Ethiopia, from June to September 2016 (N = 548)

Variables	Frequencies	Percent (%)
Residence		
Urban	311	56.8
Rural	237	43.2
Age		
15–20	46	8.4
21–25	183	33.4
26–30	194	35.4
31–35	66	12
36+	59	10.2
Marital status		
Married	538	98.2
Single	10	1.8
Religion		
Orthodox	516	94.2
Muslim	32	5.8
Educational status of the client		
Unable to read and write	162	29.6
Read and write only	62	11.3
Primary education	141	25.7
Secondary and above	183	33.4
Occupational status of the client		
Housewife	103	18.8
Gov’t employer	101	18.4
Private employee	106	19.3
Daily laborer	10	1.8
Farmer	228	41.6
Educational status of the husband		
Unable to read and write	110	20.1
Read and write only	103	18.8
Primary education	111	20.3
Secondary and above	224	40.8
Income		
≤ 1233 birr	398	72.6
> 1233 birr	150	27.4
Family size		
≤ 5	447	81.6
> 5	101	18.4

*p value < 0.05, **p value < 0.001

#### Obstetric and medical related characteristics

Majority of the respondents 511 (93.3%) had ANC follow up and three-fifth of them had at least 4 visits 326 (59.5%). Nearly all of the respondents had used modern contraceptive 488 (89%) prior to their pregnancy and majority of them had their pregnancy wanted and planned 496 (90.4%). More than two-fifth of the respondents had birth to pregnancy interval greater than or equal to 36 months 231 (42.2%). One in ten mothers had Mid Upper Arm Circumference (MUAC) less than 24 cm (10%). Labour spontaneously started in 91.6% of the respondents. One-sixth of participants had one or more obstetric complications 92 (16.8%) (Table 2).

**Table 2:** Obstetric and medical related characteristics of the women delivered in Debretabor town health institution northwest, Ethiopia, from June to September 2016 (N = 548)

Variables	Frequency	Percent (%)
MUAC		
< 24 CM	55	10
≥ 24 CM	493	90
Parity		
Primi para	233	42.5
Para 2–5	250	45.6
Para > 5	65	11.9
ANC follow up		
Yes	511	93.2
No	37	6.8
Number of ANC visit		
< 4 times	222	40.5
≥ 4 times	326	59.5
Pregnancy status		
Planned	496	90.4
Unplanned	52	9.6
Modern contraceptive use prior to current pregnancy		
Yes	488	89
No	60	11
Birth to pregnancy interval in months		
None	233	42.5
< 36	84	15.3
≥ 36	231	42.2
Poor obstetric history		
Preterm	14	2.6
Still birth	18	3.3
Abortion	101	18.4
Mode of delivery		
SVD	412	75.3
Instrumental delivery	59	10.7
C/S	77	14
Status of labor		
Spontaneous	501	91.6
Induced	33	5.9
Elective C/S	14	2.5
History of HIV testing		
Yes	523	95.6
No	25	4.4
Status of HIV		
Positive	27	4.9
Un known	25	4.6
Negative	496	90.5
Blood RH factor		
Positive	534	97.4
Negative	14	2.6
PROM		
Yes	68	12.4
No	480	87.6
HGB (mg/dl)		
< 11	52	9.5
≥ 11	496	90.5
GA at delivery		
Preterm	70	12.8
Term	415	75.7
Post term	63	11.5
Obstetric complication		
Yes	92	16.8
No	456	83.2
Type of obstetric complication		
APH	42	7.7
PIH	27	4.9
Multiple pregnancy	15	2.7
Polyhydraminous	8	1.5
History of medical illness		
Yes	60	10.9
No	488	89.1
Type of medical illness		
CHTN	13	2.4
DM	8	1.5
Heart failure	9	1.6
Asthma	5	0.9
UTI	19	3.5
Malaria	6	1.0

#### Proportion of preterm birth

The proportion of preterm birth in this study was 12.8% [95% CI (9.9%, 15.7%)] and 11.5% was post term.

#### Factors associated with preterm birth

After multivariable logistic regression, number of ANC visits less than 4 times, MUAC less than 24 cm, having PROM, being anemic, the complication in current pregnancy and being HIV positive were found to be statistically significant at a p-value of < 0.05.

Mothers who had ANC visits < 4 times in the index pregnancy were 3.3 times more likely to have a preterm birth than mothers who had ANC visits ≥ 4 times [AOR = 3.0, 95% CI (1.6–5.9)]. Mothers who had MUAC < 24 CM were 2.6 times more likely to develop preterm birth than their counterparts [AOR = 2.6, 95% CI (1.1–6.1)]. Obstetric complications during the index pregnancy were 6.6 times more likely to develop preterm birth than mothers without any of the mentioned problems [AOR = 6.6, 95% CI (3.4–12.6)]. Being HIV positive had 5.1 times increased the risk of giving preterm birth than their counterparts [AOR = 5.1, 95% CI (1.7–15.4)]. Having anemia in the index pregnancy increased the risk of preterm birth by 2.9 times [AOR = 2.9, 95% CI (1.3–6.6)] **(**Table 3).

**Table 3:** Factors associated with pre term birth among of the women delivered in Debretabor town health institution northwest, Ethiopia, from June to September 2016 (N = 548)

Variables	Preterm		COR (95% CI)	AOR (95% CI)	p-value
	Yes	No			
Residence					
Rural	42	195	2.17 (1.3–3.6)*		
Urban	28	283	1		
ANC follow up					
No	15	22	5.65 (2.8–11.5)*		
Yes	55	456	1		
Number of ANC visits					
< 4	50	172	4.4 (2.5–7.7)*	3.0 (1.6–5.9)*	0.001
≥ 4	20	306	1	1	
History of preterm delivery					
Yes	6	8	5.5 (1.8–16.4)*		
No	64	470	1		
History of abortion					
Yes	21	80	2.1 (1.2–3.7)*		
No	49	398	1		
Anemia					
Yes	17	35	4.06 (2.1–7.7)*	2.9 (1.3–6.6)*	0.01
No	53	443	1		
prom					
Yes	22	46	4.3 (2.3–7.7)*	3.0 (1.5–6.2)*	0.002
No	48	432	1	1	
MUAC (cm)					
< 24	12	43	2.09 (1.04–4.2)*	2.6 (1.1–6.1)*	0.022
≥ 24	58	435	1	1	
Complication in current pregnancy					
Yes	37	55	8.6 (4.99–14.9)*	6.6 (3.4–12.6)**	< 0.001
No	33	423	1		
Status of HIV					
Positive	12	15	6.4 (2.8–14.5)*	5.1 (1.7–15.4)*	0.003
Un known	3	22	1.0 (0.3–3.7)		
Negative	55	441	1	1	
History of medical illness					
Yes	17	43	3.2 (1.7–6.1)*		
No	53	435	1		

*p value < 0.05, **p value < 0.001

### Discussion

In this study the proportion of preterm birth was found to be 12.8% with 95% CI (9.9%–15.7%). This finding was in line with findings in Africa (11.9%) and North America (10.9%) [5], the prevalence of preterm birth in Ethiopia 10.1% [29], a study conducted in Debremarkos town health institutions 11.6% [27], and Gondar university hospital 14.3% [28].

The current finding was higher than the findings conducted in Sweden 5.03% [31], in Gondar town health institutions 4.4% [30]. This might be due to the difference in study time, setting, and design used. However, this finding was lower than findings reported in Malawi 16.3% [18], Brazil 21.7% [14], Nigeria 23.7% [24]. This variation might be because of the difference in a study area, design, time, population and sociocultural variations.

Having pregnancy complications during index pregnancy were 6.6 times more likely to have a preterm birth than mothers without these problems [AOR = 6.6, 95% CI (3.4–12.6)]. This finding was parallel with findings in Ethiopia [30], Nigeria [24] Bangladesh [22], Brazil [14] and Kenya [19]. This might be due to that obstetric complications result in medical induced or spontaneous preterm delivery.

The current study showed that having PROM were 3 times more likely to have a preterm birth than their counterparts [AOR = 3.0, 95% CI (1.5–6.2)]. This finding was similar with findings in Tehran, Iran, [32], in Kenya [19] and in Debremarkos town, Ethiopia [27]. This might be due to that labor will spontaneously initiate within hours after term PROM and within a week after preterm PROM in the majority of the cases.

In this finding being anemic were 2.9 times more likely to have a preterm birth than their counterparts [AOR = 2.9, 95% CI (1.3–6.6)]. This finding was agreed with findings in Saudi Arabia [12], in Ethiopia [27] and in Malawi [18]. This might be due to anemia predisposes to decreased blood flow to the placenta causing placental insufficiency and result in preterm delivery.

In this study, mothers with less than four times ANC visit were 3 times at risk to give preterm birth than those with four and more visits [AOR = 3.0, 95% CI (1.6–5.9)]. This finding is in line with a study in Cameron [17]. This might be frequent ANC visit maximizes the opportunity of health promotion, early detection, and treatment of obstetric complications.

The current finding showed that maternal MUAC less than 24 cm were 2.6 times increased risk of developing preterm birth than mothers with MUAC greater than or equal to 24 cm with [AOR 2.6, 95% CI (1.1–6.1)]. This finding was agreed with a study in Bangladesh [22]. This might be maternal nutritional status had a direct effect on placental size, fetus and strength of the membrane resulted in preterm delivery.

This study showed that HIV positive mothers were 5.1 times at increased risk of having a preterm birth than their counterparts [AOR = 5.1, 95% CI (1.7–15.4)]. This finding is in line with a study in Ethiopia [30]. This might be due to the drug effect and immunity of the mother at risk for preterm birth.

### Conclusion

The proportion of preterm birth was high. Problem with current pregnancy, being anemic, being HIV positive, MUAC less than 24 cm and ANC visit less than four times were found to be statistically significant for preterm birth in the current pregnancy. So that, Ministry of health need to engage on educating the community to improve ANC service utilization through different methods and Upgrade capacity of health institutions to identify treat obstetric complications and nutritional counseling for mothers during their ANC follow up.

### Limitation

This study was conducted through a cross-sectional study and may not show the cause and effect relationship.

## Abbreviations

ANC: Ante Natal Care
AOR: adjusted odds ratio
APH: ante-partum hemorrhage
CHTN: chronic hypertension
CI: confidence interval
COR: crud odds ratio
CS: cesarean section
DM: diabetes mellitus
DTH: Debretabor Hospital
EDHS: Ethiopian Demographic and Health Survey
GA: gestational age
HC: Health Center
HCT: hematocrit
HGB: hemoglobin
HIV: human immune deficiency virus
LMICs: lower and middle income countries
MDG: millennium development goal
MUAC: mid upper arm circumference
NICU: Neonatal Intensive Care Unit
PIH: pregnancy induced hypertension
PROM: premature rupture of membrane
SD: standard deviation
SVD: spontaneous vaginal delivery
SPSS: Statistical Package for Social Sciences
UTI: urinary tract infection
WHO: World Health Organization

## References

[1] Lumley J (2003). Defining the problem: the epidemiology of preterm birth. BJOG Int J Obstet Gynaecol..

[2] Blencowe H, Cousens S, Chou D, Oestergaard M, Say L, Moller A-B (2013). Born too soon: the global epidemiology of 15 million preterm births. Reprod Health..

[3] Dey AC, Mannan A, Saha L, Hossain I (2012). Review article magnitude of problems of prematurity- national and global perspective: a review. Bangladesh J Child Health.

[4] Brown HK, Speechley KN, Macnab J, Natale R, Campbell MK (2014). Neonatal morbidity associated with late preterm and early term birth: the roles of gestational age and biological determinants of preterm birth. Int J Epidemiol.

[5] Beck S, Wojdyla D, Say L, Betran AP, Merialdi M, Requejo JH (2010). The worldwide incidence of preterm birth: a systematic review of maternal mortality and morbidity. Bull World Health Organ.

[6] Johnston KM, Gooch K, Korol E, Vo P, Eyawo O, Bradt P (2014). The economic burden of prematurity in Canada. BMC Pediatr.

[7] Lawn JE, Gravett MG, Nunes TM, Rubens CE, Stanton C (2010). Global report on preterm birth and stillbirth (1 of 7): definitions, description of the burden and opportunities to improve data. BMC Pregnancy Childbirth..

[8] Marchant T, Willey B, Katz J, Clarke S, Kariuki S, ter Kuile F (2012). Neonatal mortality risk associated with preterm birth in east africa, adjusted by weight for gestational age: individual participant level meta-analysis. PLoS Med..

[9] Ministry of Health (2015). Health sector transformation plan (2015/16–2019/20).

[10] Arora CP, Kacerovsky M, Zinner B, Ertl T, Ceausu I, Rusnak I (2015). Disparities and relative risk ratio of preterm birth in six Central and Eastern European centers. Croat Med J..

[11] Rouget F, Lebreton J, Kadhel P, Monfort C, Bodeau-Livinec F, Janky E (2013). Medical and sociodemographic risk factors for preterm birth in a French caribbean population of african descent. Matern Child Health J.

[12] Alabbasi KH, Kruger E, Tennent M (2015). Maternal variables as potential modifiable risk indicators of preterm labor in Jeddah, Saudi Arabia. J Pregnancy Child Health.

[13] Demmouche A, Mai AH, Kaddouri MS, Ghani A, Rahmani S, Beddek F (2014). Etiology of preterm birth in Relizane region (West of Algeria). J Nutr Food Sci..

[14] Miranda AE, Pinto VM, Szwarcwald CL, Golub ET (2012). Prevalence and correlates of preterm labor among young parturient women attending public hospitals in Brazil. Rev Panam Salud Public..

[15] Passini R, Cecatti JG, Lajos GJ, Tedesco RP, Nomura ML, Dias TZ (2014). Brazilian multicentre study on preterm birth (EMIP): prevalence and factors associated with spontaneous preterm birth. PLoS ONE.

[16] Mokuolu OA, Suleiman BM, Adesiyun OO, Adeniyi A (2010). Prevalence and determinants of pre-term deliveries in the University of Ilorin Teaching Hospital, Ilorin, Nigeria. Pediatr Rep..

[17] Mah E, Nguefack S, Tchokoteu PF, Mah EM, Mvondo N, Nguefack S (2016). Women â€^TM^ s Health and Action Research Centre (WHARC) risk factors for premature births: a cross-sectional analysis of hospital records in a cameroonian health facility published by: Women â€^TM^ s Health and Action Research Centre (WHARC) Stable URL: htt. Women’s Health Action Res Cent..

[18] Van Den Broek NR, Jean-Baptiste R, Neilson JP (2014). Factors associated with preterm, early preterm and late preterm birth in Malawi. PLoS ONE.

[19] Wagura PM (2014). Prevalence and factors associated with preterm birth. MBChB (Moi)..

[20] Shubhada S, Kambale S, Phalke B. Determinants of preterm labour in a rural medical college hospital in western Maharashtra. Nepal J Obstet. 2013;8(1):31–3. http://www.nepjol.info/index.php/NJOG/article/view/8858.

[21] da Silveira MF, Santos IS, Barros AJD, Matijasevich A, Barros FC, Victora CG (2008). Aumento da prematuridade no Brasil: revis??o de estudos de base populacional. Rev Saude Publica.

[22] Shah R, Mullany LC, Darmstadt GL, Mannan I, Rahman SM, Talukder RR (2014). Incidence and risk factors of preterm birth in a rural Bangladeshi cohort. BMC Pediatr..

[23] Olusanya BO, Ofovwe GE (2010). Predictors of preterm births and low birthweight in an inner-city hospital in sub-Saharan Africa. Matern Child Health J.

[24] Onankpa BOIK (2014). Pattern of preterm delivery and their outcome in a tertiary hospital. Int J Health Sci Res..

[25] Iyoke CA, Lawani LO, Ezugwu EC, Ilo KK, Ilechukwu GC, Asinobi IN (2015). Maternal risk factors for singleton preterm births and survival at the University of Nigeria Teaching Hospital, Enugu, Nigeria. Niger J Clin Pract..

[26] Kunle-Olowu OE, Peterside OAO. Prevalence and outcome of preterm admissions at the neonatal unit of a tertiary health centre in southern Nigeria. Open J Pediatr. 2014;4:67–75. http://file.scirp.org/Html/9-1330299_43766.htm.

[27] Abdela Amanon TB. Preterm birth and associated factors among mothers who gave birth in Debremarkos Town Health Institutions, 2013 Institutional Based Cross Sectional Study. Gynecol Obstetric. 2015;5(292):5. http://www.omicsonline.org/open-access/preterm-birth-and-associated-factors-among-mothers-who-gave-birth-indebremarkos-town-health-institutions-2013-institutional-based-crosssectional-study-2161-0932-1000292.

[28] Adane AA, Ayele TA, Ararsa LG, Bitew BD, Zeleke BM (2014). Adverse birth outcomes among deliveries at Gondar University Hospital, Northwest Ethiopia. BMC Pregnancy Childbirth..

[29] Blencowe H, Cousens S, Oestergaard DC (2010). Lawn data from national, regional and worldwide estimates of preterm birth rate. Glob Action Rep Preterm Birth..

[30] Gebreslasie K (2012). Preterm birth and associated factors among mothers who gave birth in Gondar Town Health Institutions. Hindawi Publ Corp Adv Nurs..

[31] Cnattingius S, Villamor E, Johansson S, EdstedtBonamy A-K, Persson M, Wikstrom A-K (2013). Maternal obesity and risk of preterm delivery. J Am Med Assoc..

[32] Tehranian N, Ranjbar M, Shobeiri F (2015). The prevalence rate and risk factors for preterm delivery. J Midwifery Reprod Heal..

